# Neuromodulation effect of temporal interference stimulation based on network computational model

**DOI:** 10.3389/fnhum.2024.1436205

**Published:** 2024-09-25

**Authors:** Nafiseh Karimi, Rassoul Amirfattahi, Abolghasem Zeidaabadi Nezhad

**Affiliations:** Department of Electrical and Computer Engineering, Isfahan University of Technology, Isfahan, Iran

**Keywords:** transcranial temporal interference stimulation, brain stimulation, network computational model, multi scale model, neuromodulation

## Abstract

Deep brain stimulation (DBS) has long been the conventional method for targeting deep brain structures, but noninvasive alternatives like transcranial Temporal Interference Stimulation (tTIS) are gaining traction. Research has shown that alternating current influences brain oscillations through neural modulation. Understanding how neurons respond to the stimulus envelope, particularly considering tTIS’s high-frequency carrier, is vital for elucidating its mechanism of neuronal engagement. This study aims to explore the focal effects of tTIS across varying amplitudes and modulation depths in different brain regions. An excitatory-inhibitory network using the Izhikevich neuron model was employed to investigate responses to tTIS and compare them with transcranial Alternating Current Stimulation (tACS). We utilized a multi-scale model that integrates brain tissue modeling and network computational modeling to gain insights into the neuromodulatory effects of tTIS on the human brain. By analyzing the parametric space, we delved into phase, amplitude, and frequency entrainment to elucidate how tTIS modulates endogenous alpha oscillations. Our findings highlight a significant difference in current intensity requirements between tTIS and tACS, with tTIS requiring notably higher intensity. We observed distinct network entrainment patterns, primarily due to tTIS’s high-frequency component, whereas tACS exhibited harmonic entrainment that tTIS lacked. Spatial resolution analysis of tTIS, conducted via computational modeling and brain field distribution at a 13 Hz stimulation frequency, revealed modulation in deep brain areas, with minimal effects on the surface. Notably, we observed increased power within intrinsic and stimulation bands beneath the electrodes, attributed to the high stimulus signal amplitude. Additionally, Phase Locking Value (PLV) showed slight increments in non-deep areas. Our analysis indicates focal stimulation using tTIS, prompting further investigation into the necessity of high amplitudes to significantly affect deep brain regions, which warrants validation through clinical experiments.

## 1 Introduction

Electrical stimulation has been a crucial tool in treating various movement disorders, including Parkinson’s, Alzheimer’s, and epilepsy (Simula et al., 2022; Fox et al., 2023), as well as cognitive conditions. In addition to its therapeutic applications, electrical stimulation has significantly advanced our understanding of the complex mechanisms underlying cognition, emotion, and behavior (Polanía et al., 2018; Liu et al., 2024). Invasive electrical stimulation, particularly Deep Brain Stimulation (DBS), has proven to be an effective method for precisely targeting therapeutic sites deep within the brain. However, the invasive nature of DBS carries risks such as hemorrhage and infarction, highlighting the need to explore noninvasive alternatives in certain clinical contexts. Noninvasive Electrical Stimulation (NIES), traditionally used for superficial brain regions, has shown potential for reaching deeper targets (Huang and Parra, 2019; Vöröslakos et al., 2018). Recent studies suggest that High-Definition stimulation combined with alternating current can stimulate deep brain regions, though such stimulation is not highly focal and depends on specific conditions (Huang and Parra, 2019). Additionally, Intersectional tDCS uses brief, temporally superimposed pulses from multiple electrodes to target deep brain regions with minimal cortical stimulation by creating a pseudo-DC waveform (Vöröslakos et al., 2018).

### 1.1 Temporal interference stimulation and its challenges

The development of transcranial Temporal Interference Stimulation (tTIS) has provided an approach for modulating neural activity using kilohertz (kHz) frequency alternating current (AC) (Grossman et al., 2017). It builds on previous applications of temporal interference in peripheral nerve stimulation (Opitz and Tyler, 2017). In tTIS, pairs of electrodes generate slightly different frequencies, producing an amplitude modulation (AM) waveform that neurons respond to at the beat frequency, rather than the high-frequency components. However, tTIS faces several challenges. Despite promising results from computational models and animal studies, its effectiveness for deep brain stimulation in humans remains uncertain, primarily due to differences in brain size between humans and rodents (Lozano, 2017). While earlier studies suggested the influence of tTIS on nerve elements within the stimulation path was unclear (Lozano, 2017), more recent work has provided insights into the mechanisms of action for neural fibers and cell bodies due to tTIS (Howell and McIntyre, 2021). Additionally, tTIS is currently about 80% less effective than other non-invasive brain stimulation methods and is unlikely to induce widespread neuronal entrainment (Vieira et al., 2024). The electric fields induced by tTIS are significantly lower than those achieved with DBS, suggesting that tTIS may not produce outcomes comparable to DBS (Rampersad et al., 2019). Furthermore, realistic brain models have demonstrated that the field intensity of tTIS is more pronounced in the cortex than in deeper brain regions(Violante et al., 2023; Esmaeilpour et al., 2021; Xiao et al., 2019), underscoring the need for precise control of field parameters in NIES methodologies.

### 1.2 Mechanisms of action of tTIS

The mechanisms underlying tTIS and its efficacy in stimulating the human brain are still debated. Some researchers suggest that the low-pass filtering properties of neuronal membranes, which filter out high-frequency components, could explain why neurons respond to the envelope of modulated signals (Grossman et al., 2017). However, this explanation is challenged by the fact that amplitude modulation (AM) signals, which primarily contain high-frequency components, should not be effectively filtered out. Suprathreshold models, such as those reported by (Mirzakhalili et al., 2020; Howell and McIntyre, 2021), propose that neurons respond to the modulated signal envelope through a demodulation process facilitated by rectification mechanisms. This involves the nonlinear response of axonal fibers and aligns with experimental findings describing activation due to an integrator-threshold mechanism (Budde et al., 2023). However, recordings of muscle activity in the plantar muscles and biceps femoris indicate that interference techniques do not alter recruitment in regions far from the electrodes, and stimulation efficacy diminishes in deeper regions compared to areas closer to the electrodes. This complicates the targeting of small dimensions. These models suggest that tTIS can produce a range of effects, including phasic activation, tonic activation, quiescence, or conduction block, which challenges the selectivity of tTIS (Mirzakhalili et al., 2020; Howell and McIntyre, 2021; Budde et al., 2023). On the other hand, network models and studies examining subthreshold mechanisms propose that the effects of tTIS are mediated through network resonance phenomena and interactions with endogenously oscillating systems (Esmaeilpour et al., 2021; Howell and McIntyre, 2021). These models suggest that tTIS can influence synaptic currents and network dynamics, offering a different perspective on how stimulation may modulate neuronal activity beyond the rectification hypothesis.

The literature on tTIS highlights various mechanisms, including suprathreshold and subthreshold models, emphasizing the need to understand these mechanisms to select appropriate neuron models for accurate simulation. While non-linearities in ion channel-based models, such as the Hodgkin-Huxley (HH) model, offer advantages in tTIS stimulation modeling (Xiao et al., 2019; Cao et al., 2020; Mirzakhalili et al., 2020; Howell and McIntyre, 2021), they also present certain complications. Simpler models like the Izhikevich (IZI) model are more suitable for studying collective neuronal behavior due to their reduced complexity (Reato et al., 2010; Esmaeilpour et al., 2021; Budde et al., 2023), with further experiments needed to refine these models.

### 1.3 Exploring neuromodulation effects

The neuromodulation effects of tTIS have been investigated by stimulating occipitoparietal alpha oscillations and the primary motor cortex using 20 Hz and 70 Hz beat frequencies in human participants (Ma et al., 2021; von Conta et al., 2022). Ma et al. demonstrated that tTIS could serve as a valuable tool for exploring the specific roles of different brain oscillations in various cognitive tasks, particularly those originating from deep brain regions. Their results suggest that tTIS might operate through a low-frequency envelope mechanism, evidenced by its distinct effects on various motor tasks using different envelope frequencies. While Ma’s study highlighted the feasibility of tTIS as a stimulation tool in humans, von Conta et al. found no significant differences in the effects of tTIS compared to tACS and a control group. This finding implies either uniform stimulation across all groups or a lack of effective stimulation. The presence of a 1 kHz stimulation signal in the control group highlights the need to explore stimulation effects at higher frequencies. Both studies faced challenges in demonstrating the efficacy of tTIS at a human scale and in comparing its effects to conventional tACS. They also investigated the impact of tTIS beat frequency through experimental research. It is crucial to explore the mechanisms of tTIS at the level of brain regions and networks, examine the effects of carrier and envelope frequencies more deeply, and strive to develop a more effective tTIS stimulation system for humans.

### 1.4 Computational network models

To address these challenges, computational network models offer a promising approach. Network models, such as the Excitatory-Inhibitory (E-I) model, allow for detailed investigation of neural interactions and can simulate complex neural dynamics that are difficult to capture experimentally (Reato et al., 2010; Ali et al., 2013; Huang et al., 2021). For example, the E-I network model can explore how different frequencies and intensities of tTIS affect neural activity across various brain regions.

Neural target engagement through amplitude-modulated tACS (AM-tACS) was investigated using an E-I network (Negahbani et al., 2018). The carrier frequency was less than 200 Hz, significantly differing from the range used in tTIS. The findings suggest that higher carrier frequencies require greater intensity for effective stimulation. Additionally, tTIS’s ability to entrain gamma oscillations has been demonstrated using computational models and experimental research on mouse hippocampal slices (Esmaeilpour et al., 2021). However, computational models employing the E-I network with Adaptive Exponential Integrate-and-Fire (Adex) neurons, along with human brain models based on MRI data, indicate that tTIS struggles to stimulate deep brain targets in a suprathreshold manner. In these models, the overlying cortex responds to static rather than dynamic modulation in deeper regions (Esmaeilpour et al., 2021). These results are consistent with other computational studies confirming tTIS’s limited effectiveness in stimulating deep brain targets suprathresholdly (Mirzakhalili et al., 2020; Howell and McIntyre, 2021). Also, Esmaeilpour et al. examined tTIS sensitivity and selectivity concerning gamma oscillations, focusing on biological factors like the neuronal membrane time constant and GABA_*b*_ neurotransmitters. Their study specifically addressed a stimulation frequency of 5 Hz and a limited intensity range. Present research extends this investigation by exploring a broader parameter space for input stimuli, focusing on amplitude and stimulation frequency, and reveals Arnold tongue features—regions in the parameter space where synchronization occurs between neural activity and stimulation frequency. Our approach aims to identify the threshold for stimulation intensity across various frequencies to achieve neural entrainment, with a particular emphasis on alpha oscillations. We analyze neural entrainment using criteria such as Phase-Locking Value (PLV), which measures the consistency of phase differences between neural signals and stimulation, as well as amplitude and frequency entrainment. This approach provides a more nuanced understanding of the effects of transcranial temporal interference stimulation. By identifying these parameters, our work broadens the perspective of previous studies, including those by Esmaeilpour et al., and aims to enhance the application of tTIS in neuromodulation.

### 1.5 Study objectives

The primary objective of this study is to elucidate the key factors influencing stimulation through the use of a computational network model, aiming to gain deeper insights into the parameters associated with tTIS. This approach highlights the relative differences in the mechanisms of action between tTIS and tACS, providing a clearer understanding of how tTIS interacts with neural networks. Additionally, a macroscopic brain model is employed in conjunction with COMSOL to compute the distribution of the electric field. By integrating data from our computational network model with the macroscopic model, we aim to identify the stimulated area based on the parameters involved in modulating neural activity within the brain.

## 2 Materials and methods

### 2.1 Bioelectric field models in a spherical head model

To characterize the extracellular field distribution generated during temporal interference stimulation, two sinusoidal currents with frequencies f1 and f2 are applied to the first and second electrode pairs, respectively. The total electric field is calculated by summing up the ones generated by each electrode pair:


$$E_{t\,o\,t\,a\,l}\left(x.y.z.t\right)=E_{1}\left(x.y.z\right)sin\left(2\pi f_{1}t\right)+E_{2}\left(x.y.z\right)sin\left(2\pi f_{2}t\right)$$

Where E1 and E2 are the electric field produced by the first and second pair of electrodes, respectively. Assuming quasi-static current conditions, the electric potential is calculated using Laplace equation (Cheng, 1989):


∇⁡⋅⁢(σ⁢∇⁡Φ)=0

Here, σ represents tissue conductivity, Φ is electric potential and ∇ is the gradient operator. The electric field is derived from the computed electric potential as follows:


$$\vec{\text{E}}=-\nabla \Phi$$

$$\vec{J}=\sigma \,\vec{\text{E}}$$

To solve Laplace equation, a simplified single-layered head model is established in COMSOL by utilizing the coefficient form partial differential equation (PDE) Physics module. This model is employed to compute the induced current densities produced by each electrode set, utilizing the Finite Element Method (FEM).

Based on equation 1, the total electric field forms an AM modulated signal, and the maximum envelope amplitude (modulation depth) along any direction in $\vec{r}\left(x.y.z\right)$ is obtained as follows (Grossman et al., 2017):


$$\left|{\vec{E}}_{A\,M}^{m\,a\,x}\,\left(\vec{r}\right)\right|=$$

$$\left\{ \begin{array}{c} 2\,\left|{\vec{E}}_{2}\,\left(\vec{r}\right)\right|\;i\,f\,\left|{\vec{E}}_{2}\,\left(\vec{r}\right)\right|<\left|{\vec{E}}_{1}\,\left(\vec{r}\right)\right|\,c\,o\,s\,\left(\alpha \right)\\ \frac{2\,\left|{\vec{E}}_{2}\,\left(\vec{r}\right)\times \left({\vec{E}}_{1}\,\left(\vec{r}\right)-{\vec{E}}_{2}\,\left(\vec{r}\right)\right)\right|}{\left|{\vec{E}}_{1}\,\left(\vec{r}\right)-{\vec{E}}_{2}\,\left(\vec{r}\right)\right|}\;o\,t\,h\,e\,r\,w\,i\,s\,e \end{array}\right.$$

Where α < π/2 is the angle between ${\vec{E}}_{1}$ and ${\vec{E}}_{2}$. It is also assumed that $\left|{\vec{E}}_{1}\right|$ is greater than $\left|{\vec{E}}_{2}\right|$.

A single-layer sphere with a constant conductivity of 0.333 S/m and diameter of 18 cm is employed to model the spatial distribution of the electric field within human brain tissue. Electrodes are 1 mm in height and 8 mm in radius, and are positioned in xy plane as follows:

1-The first pair of electrodes was situated at r = 9 cm, θ = 90°, with φ_1_ = −30° and φ_2_ = 30°.
2-The second pair of electrodes was situated at r = 9 cm, θ = 90°, with φ_1_ = 150° and φ_2_ = 210°.

**FIGURE 1:**
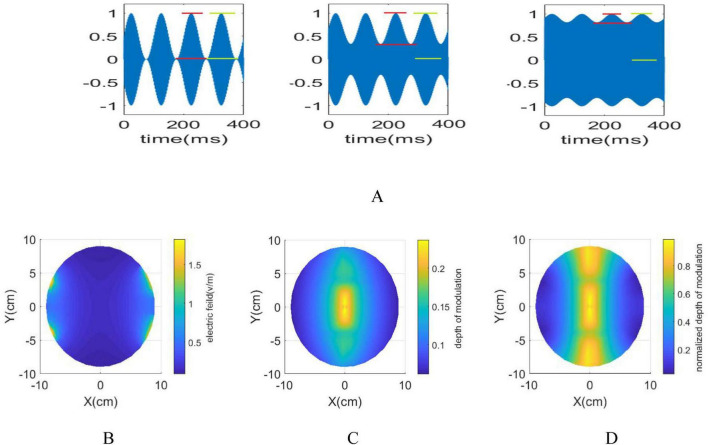
**(A)** The red lines illustrate the envelope amplitude, while the green lines represent the amplitude of the modulated signal. By moving away from the interference center towards the electrodes, the envelope amplitude decreases while the amplitude of the modulated signal increases. **(B)** $||{\vec{E}}_{2}\,\left(\vec{r}\right)||+||{\vec{E}}_{1}\,\left(\vec{r}\right)||$ in an xy plane with applying 1 mA current to each electrodes **(C)**. $\left|{\vec{E}}_{A\,M}^{m\,a\,x}\,\left(\vec{r}\right)\right|$ is maximum in the i interference center. By moving away from the interference center, envelope amplitude decreases **(D)**. $\frac{\left|{\vec{E}}_{A\,M}^{m\,a\,x}\,\left(\vec{r}\right)\right|}{||{\vec{E}}_{2}\,\left(\vec{r}\right)||+||{\vec{E}}_{1}\,\left(\vec{r}\right)||}$ ranges between 0 and 1, reaching 1 when the amplitude of the modulated signal aligns with the envelope amplitude. When moving along the y-direction, the ratio initially decreases, then increases. However, as approaching the electrodes from the interference center, it consistently decreases.

Although the interference center exhibits maximum envelope amplitude, the amplitude of the modulated signal is higher near the electrodes. Upon applying a 1 mA current to each pair of electrodes, the electric fields, maximum envelope amplitude and the normalized maximum envelope amplitude, defined as ($N\_\,M\,D=\frac{\left|{\vec{E}}_{A\,M}^{m\,a\,x}\,\left(\vec{r}\right)\right|}{||{\vec{E}}_{2}\,\left(\vec{r}\right)||+||{\vec{E}}_{1}\,\left(\vec{r}\right)||}$), delineate distinct surface features (Figure 1). The regions surrounding the electrodes display low modulation depth and high amplitude, while the interference center demonstrates maximum modulation depth but comparatively lower amplitude. The similarity in the ratio between the maximum envelope modulation and the stimulus signal amplitude is observed both within the interference center and its surrounding regions. To effectively determine whether nerve elements along the stimulation path respond and to understand potential side effects, the amplitude of the stimulation signal on the electrodes is gradually increased until the stimulation effect is noticeable within the target area. The effectiveness of the stimulation in regions outside the target area is then evaluated based on specific predefined criteria.

Furthermore, Studies exploring non-invasive electrical stimulation highlight the amplitude of the stimulation signal as a crucial factor influencing its effectiveness(Liu et al., 2018; Bland and Sale, 2019). However, investigations employing tissue modeling to assess stimulation effectiveness often establish a threshold, typically 0.2 V/m of the maximum modulation depth, as indicative of optimal stimulation (Rampersad et al., 2019; Karimi et al., 2019; Budde et al., 2023). Notably, these studies commonly overlook the impact of high frequency in tTIS stimuli. Therefore, incorporating parameters such as stimulation amplitude and high frequency alongside modulation depth into the tTIS stimulus signal using a network consisting of neurons and their connections can offer a more comprehensive understanding of tTIS efficiency.

### 2.2 Neuron model

The Excitatory-Inhibitory (E-I) network represents a pivotal mechanism for generating brain rhythms. Brain rhythms constitute concurrent patterns of neural activity, holding a substantial influence over diverse cognitive and behavioral processes. The E-I network embodies an intricate interplay involving excitatory and inhibitory neurons, neurotransmitters, receptors, ion channels, and other contributing factors. In the context of creating E-I networks, we assembled a configuration comprising excitatory neurons characterized by type I membrane properties and inhibitory neurons exhibiting type II membrane properties (Izhikevich, 2007). The choice of the phenomenological neuron model developed by Izhikevich was motivated by its remarkable capability to replicate a wide spectrum of electrical behaviors observed in real neurons (Izhikevich, 2003). This versatility is achieved by manipulating only a few parameters. Notably, this neuron model has been previously applied in transcranial brain stimulation (Huang et al., 2021; Ali et al., 2013; Reato et al., 2010). In essence, the neuronal dynamics are described by a pair of interlinked differential equations governed by four parameters (designated as ‘a’ through ‘d’) that determine intrinsic excitability.

The process for updating the membrane potential of each neuron adheres to the following computational rule:


$$C\frac{dv}{dt}=k\left(v-v_{r}\right)\left(v-v_{t}\right)-u+\text{I}$$

$$\frac{du}{dt}=a\left\{b\left(v-v_{r}\right)-u\right\}$$

$$ifv\;\geq \;v_{peak}thenv\;\leftarrow cu\;\leftarrow \;u+d$$

Within this context, v corresponds to the neuron’s membrane potential, while u serves as an auxiliary variable signifying the activation state of sodium and potassium currents. Auxiliary variable u introduces a positive feedback loop that influences v. Additionally, C represents the membrane capacitance, *v_r_* denotes the resting membrane potential and *v_t_* signifies the intrinsic threshold potential. The constant ‘a’ characterizes the recovery rate, while b determines the sensitivity of the auxiliary variable to changes in the membrane potential. The *v*_*peak*_ denotes the threshold voltage, c is voltage reset value, and d signifies the reset value of the auxiliary variable. Quantity I represents the summation of currents entering a neuron.

For a regular spiking pyramidal cell as an excitatory neuron (Izhikevich, 2007):

C = 100 *pf*, k = 0.7, *v*_*r*_ = −60 mv, v_*t*_ = −40 mv, a = 0.03, b = −2, v_*peak*_ = 35 mv, c = −50, d = 100 and for fast spiking inhibitory interneurons:

C = 20 *pf*, k = 1, v_*r*_ = − 55 mv, v_*t*_ = − 40 mv, v_*peak*_ = 25 mv and c = − 50.

Updating rule for auxiliary variable in an inhibitory neuron is:


$$\frac{d\,u}{d\,t}=0\cdot 2\,\left\{\text{U}\,\left(\text{v}\right)-u\right\}\;U\,\left(\text{v}\right)=\left\{ \begin{array}{c} 0\;v<-55\\ 0\cdot 025\,{\left(v+55\right)}^{3}\;o\,t\,h\,e\,r \end{array}\right.$$

### 2.3 Model of synaptic dynamics

Synapses are characterized through an exponential profile represented as:


$$g\,\left(t\right)=g_{0}*\exp\,\left(-\frac{\text{t}}{\tau }\right)$$

With g(t) representing the synaptic conductance at time t, g0 indicating the initial electrical conductance, and τ representing the time constant for this channel. Following the firing of each neuron, the electrical conductivity of the synaptic channel connecting that neuron to its counterparts reaches its maximum value (g0) and subsequently decreases over time with the time constant τ (Gerstner et al., 2014). Every neuron, regardless of whether it is excitatory or inhibitory, receives both inhibitory and excitatory synaptic currents. As a result, the total synaptic current, denoted as *I*_*Syn*_PY/FS__, is divided into distinct excitatory and inhibitory components, where the inhibitory component arises from inhibitory synapses utilizing the *GABA*_A_ neurotransmitter, and the excitatory one originates from excitatory synapses utilizing the AMPA neurotransmitter. Thus, the synaptic current can be expressed as follows:


$$I_{S\,y\,n_{\mathrm{PY}/\mathrm{FS}}}\,\left(t\right)=\text{G}\,\left(t\right)\,\left(\text{V}\,\left(t\right)-E\right)$$

Where V(t) is the membrane potential of the neuron at time t, G(t) is the sum of the electrical conductivity of inhibitory/excitatory synapse which is defined by one of the following equations:


$$G_{E\,E}\,\left(t\right)=\sum_{\begin{array}{c} \text{i}=\text{excitatory}\,\text{neurons} \end{array}}g_{0_{E\,E}}\,\exp\,\left(-\frac{t-t_{f_{i}}}{\tau_{E}}\right)$$

$$G_{I\,E}\,\left(t\right)=\sum_{\begin{array}{c} \text{i}=\text{inhabitory}\,\text{neurons} \end{array}}g_{0_{I\,E}}\,\exp\,\left(-\frac{t-t_{f_{i}}}{\tau_{I}}\right)$$

$$G_{E\,I}\,\left(t\right)=\sum_{\begin{array}{c} \text{i}=\text{excitatory}\,\text{neurons} \end{array}}g_{0_{E\,I}}\,\exp\,\left(-\frac{t-t_{f_{i}}}{\tau_{E}}\right)$$

$$G_{I\,I}\,\left(t\right)=\sum_{\begin{array}{c} \text{i}=\text{inhabitory}\,\text{neurons} \end{array}}g_{0_{I\,I}}\,\exp\,\left(-\frac{t-t_{f_{i}}}{\tau_{I}}\right)$$

Where *t*_*f_i_*_ is the firing time of i-th neuron. It should be noted that the above relationship is valid for *t* > *t*_*f*_ and the synaptic conductance considered to be zero for *t* < *t*_*f*_. The maximum conductance values for each synapse are *g*_*0_EE_*_ = 0.3, *g*_*0_IE_*_ = 0.3, *g*_*0_EI_*_ = 0.4 and *g*_*0_II_*_ = 0.03. The time constant is τ_*E*_ = 2ms for excitatory synapses, and τ_*I*_ = 10ms for inhibitory synapses(Gerstner et al., 2014). *I*_*Syn*_PY/FS__, entering each inhibitory or excitatory neuron, is defined as:


$$I_{s\,y\,n.P\,Y}\,\left(t\right)=G_{E\,E}\,\left(t\right)\,\left(E_{A\,M\,P\,A}-V_{P\,Y}\,\left(t\right)\right)+$$

$$G_{I\,E}\,\left(t\right)\,\left(E_{G\,A\,B\,A_{A}}-V_{P\,Y}\,\left(t\right)\right)$$

$$I_{s\,y\,n.F\,S}\,\left(t\right)=G_{E\,I}\,\left(t\right)\,\left(E_{A\,M\,P\,A}-V_{F\,S}\,\left(t\right)\right)+$$

$$G_{I\,I}\,\left(t\right)\,\left(E_{G\,A\,B\,A_{A}}-V_{F\,S}\,\left(t\right)\right)$$

In this regard, *E_AMPA_* = 0 *mv* and *E*_*GABA*_A__ = − 70 *mv* are the reverse potential of the excitatory and inhibitory synapse while *V*_PY/FS_(*t*) denotes the membrane potential of the neuron at time t.

### 2.4 Network topology

The network comprises 80 excitatory and 20 inhibitory neurons, maintaining an excitatory-to-inhibitory ratio of 4:1, consistent with cortical observations (Markram et al., 2004). While previous work (Esmaeilpour et al., 2021) utilized 1000 neurons, our study employs a total of 100 neurons. This choice is justified because it effectively captures the dynamics between excitatory and inhibitory neurons and models crucial neural interactions, as demonstrated by (Huang et al., 2021), while also requiring significantly fewer computational resources.

**FIGURE 2:**
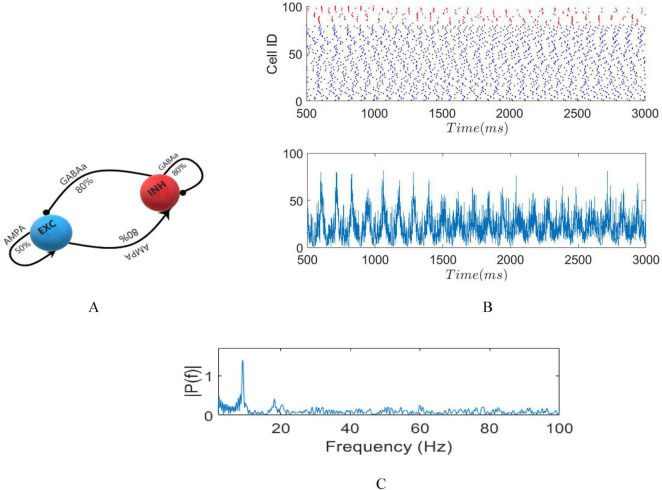
**(A)** Diagrammatic representation of the network. **(B)** raster plot in absence of stimuli (top) LFP signal (bottom). **(C)** Fourier transform of LFP with dominant frequency of 9 Hz.

Neurons establish connections randomly, following a uniform probability represented as *p*_*ij*_. The probability of connection between a postsynaptic neuron (j) and a presynaptic neuron (i) depends on the specific types of both neurons involved. Therefore, *p*_*EE*_ = 0.5, where E represents excitatory neurons, and each excitatory neuron can potentially connect uniformly to another excitatory one (global connection). Conversely, *p*_*EI*_ = 0.8 and *p*_*IE*_ = 0.8, where I denotes inhibitory neurons, each able to form synaptic connections with 32 adjacent excitatory neurons, with connection probability of 80%, as established for E-I connections (local connection). Furthermore, *p*_*II*_ = 0.8, indicating that each inhibitory neuron establishes synaptic connections with 10 adjacent inhibitory neurons within the inhibitory network with an 80% probability (local connection). This choice of connectivity density aligns with previous research (Harris and Shepherd, 2015; Naka and Adesnik, 2016). Diagrammatic representations of these networks are displayed in Figure 2A.

To induce spontaneous oscillations in the network, we apply a DC current of 57 pA to the pyramidal neurons and 68 pA to the inhibitory neurons. These specific DC currents were chosen by adjusting the DC current for the pyramidal neurons from 55 to 63 pA and for the inhibitory neurons from 65 to 70 pA in 0.5 pA increments. This tuning aimed to achieve a dominant frequency of 9 Hz, corresponding to the alpha band, in the Local Field Potential (LFP). This setup ensures that interneurons do not generate action potentials unless they receive input from excitatory cells. Subsequently, we will investigate the effects of tTIS on these intrinsic network oscillations. The injected current into each excitatory neuron, as defined in equation (16), is:


$$I=I_{d\,c}+I_{s\,y\,n}+I_{s\,t\,i\,m}+I_{n\,o\,i\,s\,e}$$

Where *I*_*stim*_ represents the current entering the neuron due to stimulation and is considered as:


$$I_{s\,t\,i\,m}\,\left(t\right)=\text{A}\,s\,i\,n\,\left(2\,\pi \,f_{1}\,t\right)+B\,s\,i\,n\,\left(2\,\pi \,f_{2}\,t\right)$$

Where *f*_1_ = *f*_C_ + *f*_m_, *f*_2_ = *f*_C_ with *f*_C_ set at 1 kHz while *f*_m_ will be adjusted for investigation purposes. Moreover, *A* = *B* in the interference center and the modulation depth reaches its maximum. Additionally, *I*_*noise*_ is a zero mean Gaussian noise with standard deviation of 0.5 pA. The injected current into each inhibitory neuron mirrors that of the excitatory neurons, while *I*_*stim*_ is exclusively directed to excitatory neurons due to their distinct morphology (Radman et al., 2009).

A raster plot representing the firing activity of both excitatory and inhibitory neurons in the absence of external stimuli along with the corresponding LFP and spectrum, is depicted in Figures 2B, C.

### 2.5 Measures

Local field potentials are the result of concurrent neural activity within a specific region of the brain. The mean absolute value of presynaptic currents entering pyramidal neurons is calculated as LFP (Mazzoni et al., 2008):


$$\text{LFP}=\sum_{i=1}^{N}\frac{\left|I_{A\,M\,P\,A.i}\right|+\left|I_{G\,A\,B\,A_{A}.i}\right|}{N}$$

In this context, I_*AMPA*_ represents the excitatory synaptic current entering the i-th neuron, while *I*_*GABA_A_*_ denotes the inhibitory synaptic current received by the neuron and N represents the total number of neurons.

Multiple approaches are utilized to quantify the dynamics of network activity, with entrainment serving as a primary criterion for computing network dynamics. Entrainment, a phenomenon observed when neural activity synchronizes or becomes phase-locked to external rhythmic stimulation, encompasses frequency modulation, amplitude modulation and phase synchronization (Vosskuhl et al., 2018). Frequency modulation occurs when the frequency of external stimulation matches or closely aligns with the natural frequency of neural oscillations in the brain region of interest. Phase entrainment, on the other hand, involves aligning the phase of the LFP signal with the phase of the external stimulus. This synchronization is often assessed using phase coherence or phase-locking value. Phase Lock Value (PLV) is determined by subtraction of instantaneous phase of stimulation signal and LFP (Lachaux et al., 1999). This instantaneous phase is extracted by Hilbert transform.


PLV=1N|∑n=1Ne|j⁢(φA⁢(n)-φB⁢(n))

Where φ_*A*_(*n*) and φ_*B*_(*n*) are the instantaneous phase stimulation signal and LFP, respectively. According to above equation, PLV is between 0 and 1 and it is equals 1 when the stimulation signal and LFP are perfectly phase-locked (Aydore et al., 2013).

Moreover, the power of stimulation frequency band, attributed as amplitude entrainment, serves as a third criterion alongside frequency and phase entrainment.

### 2.6 Integration of spherical head model and network model

The electric fields calculated in COMSOL are transformed to intracellular injection current (*I*_*stim*_) using F as a scale factor (Xiao et al., 2019):


$$F\cdot \frac{1}{\pi \,D\,L}\cdot I_{s\,t\,i\,m}=J$$

Where *J* = σE with *D* =  9.6μm and *L* =  9.6μm as the diameter and length of soma, respectively. For instance, an electric field equal to 23 V/m induces *I*_*stim*_ =  2200 *pA*. Consequently, the amplitude of the modulated signal will be 46 V/m. Thus, by injecting *I*_*electrode*_ = 63.5 mA into each pair of electrodes, the modulated electric field reaches a maximum amplitude of 46 V/m at the interference center (modulation depth = 1), as computed using the FEM model.

To aggregate the results of the network model and the spherical head model, the required applied current to the electrodes (*I*_*electrode–th*_) is determined. *I*_*electrode–th*_ is proportional to the minimum stimulation current (*I*_*stim–th*_) which is needed to induce a significant change in network synchrony in center of interference, based on metrics derived from LFP. Electric fields and modulation depth (*E*_1_, *E*_2_ and ${\vec{E}}_{A\,M}^{m\,a\,x}$) are calculated by applying *I*_*electrode–th*_ to each electrodes. The temporal stimulation signal is created with respect to f1 and f2 and based on Eq.1. The equivalent current intensity is calculated using Eq.20 and *I*_*stim*_ is then provided as the stimulation current to excitatory neurons in each network. The networks are situated within the brain on the x-y plane, characterized by a resolution of 6 mm in the radial direction and 0.06π rad in the azimuthal direction in polar coordinates. Following this positioning, LFP is computed for each network, and the pertinent metrics are determined. The stimulated area, based on the mentioned criteria, will determine the focality of tTIS.

## 3 Results

tTIS is recognized for its potential as a noninvasive method for altering current stimulation that can effectively target deeper regions within the brain. This paper primarily explores the initial neuromodulatory effects of tTIS. To achieve this objective, we implemented both unmodulated and modulated stimulation methodologies. Our goal was to investigate whether the neuromodulatory effects of tTIS align with those of tACS. To explore this, a sinusoidal signal with the frequency similar to the beat frequency of tTIS (*f_m_*) was generated as the stimulation current and was given to the neuronal network. Subsequently, we created a modulated stimulation signal by summing two sinusoidal signals (Eq. 17), both sharing the same amplitude where*f*_2_ = 1 kHz and *f*_1_ = *f*_2_ + *f*_*m*_. This comparison has also been employed in studies on alpha band stimulation in human research (Huang et al., 2021) and brain slice stimulation involving intrinsic gamma oscillations (Esmaeilpour et al., 2021). Exploring variations in *f_m_* and stimulation amplitude allowed us to delineate parameter space.

### 3.1 tACS stimulation

To investigate the impact of stimulation, a sinusoidal signal with amplitude ranging from 0 to 60 pA and frequencies varying between 5 and 35 Hz was applied to the network. Subsequently, the LFP signal was estimated during each stimulation, enabling the extraction of the specified features.

**FIGURE 3:**
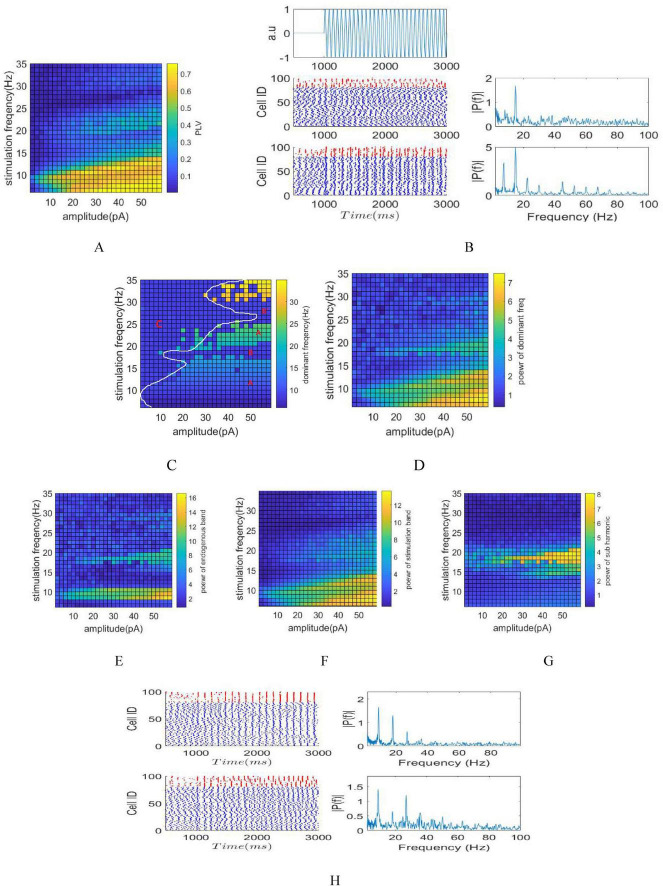
Stimulation of the network by AC signals with varying amplitude and frequency. **(A)** Phase entrainment heat map by changing amplitude ranging from 0 to 60 pA with stimulation frequencies varying between 5 and 35 Hz. **(B)** raster plot and Fourier transform of the LFP signal corresponding to stimulations with currents of 20 and 50 pA and stimulation frequency of 15 Hz. Phase, amplitude and frequency entrainments are more obvious with 50 pA stimulation due to its larger amplitude, the Fourier transform of the LFP of 50 pA stimulation shows subharmonic frequency components (7.5 Hz) during stimulation. **(C)** Frequency entrainment heat map shows different zones based on its different frequency entrainments. **(D)** Power at dominant frequency of LFP’s. This parameter delineates the fluctuations in dominant frequency power concerning various parameters subsequent to achieving frequency entrainment. **(E)** Amplitude entrainment quantified through power computation within the intrinsic oscillation band. **(F)** Amplitude entrainment quantified through power computation within the stimulation band. **(G)** Power calculation on the subharmonic frequency band of the stimulation frequency. **(H)** Top: Raster plot and FFT of LFP for a stimulation frequency of 18 Hz and an amplitude of 40 pA (region B). Bottom: Raster plot and FFT of LFP for a stimulation frequency of 27 Hz and an amplitude of 80 pA (region B).

The illustrations in Figures 3A, C–F, G, depict phase, amplitude, and frequency entrainment due to tACS, respectively. The presence of Arnold tongue within these visuals symbolizes system synchronization and coupling, showcasing tissue responsiveness to external stimuli. Such insights, notably in applications like DBS, assist in precise targeting during stimulation, mitigating unintended effects (Zamora et al., 2021). Also, Comprehending Arnold tongue aids in optimizing parameter space, providing valuable insights into the system’s resonance capabilities (Vogeti et al., 2022).

Phase entrainment becomes apparent in smaller amplitude stimuli when the stimulation frequency aligns with the intrinsic frequency of the network, showcasing synchronization within the network. Furthermore, resonance occurs when the stimulation frequency corresponds to the second harmonic of the intrinsic frequency, depicted as the second Arnold tongue. As the stimulation frequency moves away from the intrinsic frequency, greater amplitude is necessary to induce phase entrainment. The application of a 2 pA stimulation current resulted in an increase in the PLV at the stimulation frequency of 9 Hz (equal to *f*_*ins*_). The raster plot and Fourier transform of the LFP signal are depicted in Figure 3B, corresponding to stimulation currents of 20 and 50 pA with a stimulation frequency of 15 Hz. The choice of a 15 Hz stimulation frequency is aimed at exhibiting frequency entrainment, while selecting 50 pA over 20 pA is intended to demonstrate amplitude entrainment. Beside phase entrainment, both stimulations exhibit frequency entrainment, causing a shift in the dominant LFP frequency from 9 Hz to 15 Hz during stimulation. Furthermore, the LFP power at 15 Hz in the 50 pA amplitude stimulation surpasses that of the 20 pA amplitude, indicating amplitude entrainment. Moreover, the increase in stimulation amplitude from 20 to 50 pA resulted in the emergence of subharmonic frequency components (7.5 Hz) observed in the Fourier transform of the LFP shown in Figure 3B.

The representation of frequency entrainment is illustrated through the dominant frequency of LFP (Figure 3C) and the power at this dominant frequency (Figure 3D). Observing the dominant frequency allows for distinguishing three modes: non-frequency entrainment (indicated by C), frequency entrainment aligned with the stimulation frequency (indicated by A), and frequency entrainment occurring when the stimulation frequency aligns with the harmonic of the intrinsic frequency (indicated by B). The demarcation provided by the white border roughly separates the non-entrainment zone from the entrainment zone. Additionally, the boundary between areas B and C is shown in Figure 3E, where calculation of the intrinsic power band (8–10 Hz) has been considered.

In region B, the close correspondence between the stimulation frequency and the inherent oscillation frequency (or its harmonics, specifically 18 and 27 Hz) of the network induced resonance. Consequently, the dominant LFP frequency persisted at the network’s intrinsic frequency (9 Hz) rather than aligning with the stimulation frequency. The raster plot and FFT of LFP for stimulation frequencies of 18 Hz and 27 Hz in Figure 3H demonstrate network resonance, as evidenced by aligning the raster plot with the 9 Hz stimulation frequency rather than at 18 and 27 Hz, which is more apparent in Figure 3H-Top. Additionally, the dominant LFP frequency remains at 9 Hz in both cases.

As a result, the impact of stimulation was characterized by amplitude entrainment and increased power within the intrinsic power band, rather than the dominant LFP frequency matching the stimulation frequency. Hence, distinguishing between region B and region C can be achieved by calculation of the power within the intrinsic band (Figure 3E).

To enhance comprehension, power calculation has been focused on the subharmonic frequency of the stimulation, as depicted in Figure 3G. For instance, when the stimulation frequency is 15 Hz, power is calculted within the 7.5 Hz band; similarly, at a stimulation frequency of 18 Hz, power is calculted within the 9 Hz band. The Arnold tongue depicted in this figure highlights that applying stimulation at a frequency equivalent to the harmonic of the inherent frequency results in amplitude entrainment at inherent frequency.

Additionally, examining power within the stimulation frequency band (Figure 3F) reveals the amplitude entrainment induced by tACS, complementing the assessment within the intrinsic oscillation and subharmonic bands.

### 3.2 tTIS stimulation

**FIGURE 4:**
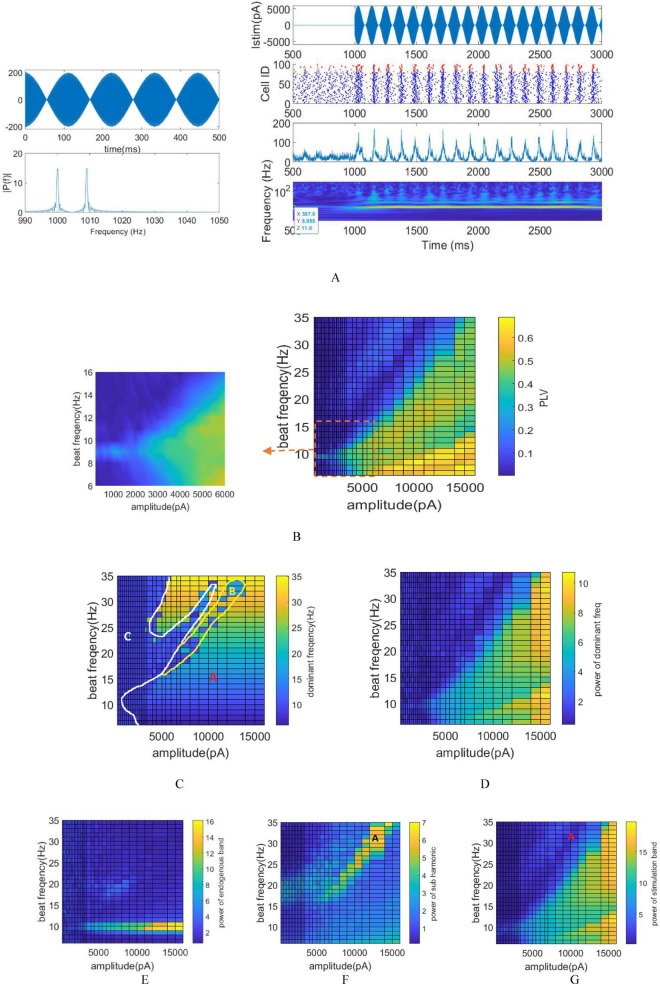
Stimulation of the network using varying amplitudes and frequencies via tTIS. **(A)** Left: Stimulation of signal and its FFT using a stimulation frequency of 9 Hz. Right: Raster plot, LFP signal, and STFT of the LFP signal corresponding to the stimulation frequency of 9 Hz and a modulated stimulation signal amplitude of 6,000 pA. **(B)** Phase entrainment heatmap exhibiting changes in stimulation signal amplitude from 200 to 16,000 pA and beat frequencies (fm) ranging between 5 and 35 Hz. The PLV heat map is magnified around the intrinsic frequency for detailed observation. **(C)** Frequency entrainment depicted by calculating the dominant frequency of LFP. **(D)** Power within the dominant frequency band. **(E)** Amplitude entrainment: Power within the intrinsic frequency band. **(F)** Amplitude entrainment: Power of the subharmonic of the stimulation frequency. **(G)** Amplitude entrainment: Power within the stimulation frequency band.

To explore the neuromodulatory effects of tTIS, the amplitude of the stimulation signal ranged from 200 to 16,000 pA, which equivalently adjusts the amplitude between 100 pA and 8000 pA for each pair of electrodes. Throughout this investigation, the assumption is that an equal current is administered to each electrode while situating the network at the interference center (modulation depth = 1), as depicted in Figure 4A. The stimulation signal adopts a sinusoidal modulation generated from the summation of two sinusoids with f1 and f2 frequencies. The beat frequency *f_m_* varies within the range of 5 to 35 Hz. The raster plot, LFP signal, and STFT of the LFP signal, corresponding to the stimulation frequency of 9 Hz and a modulated stimulation signal amplitude of 6000 pA are depicted in Figure 4A. Notably observed in the STFT plot is a clear increase in intrinsic power band during the stimulation period, attributed to the alignment between the intrinsic and beat frequency. This observation signifies that the stimulation process results in amplitude modulation. The LFP signal and the raster plot show the synchronization of the neurons activity with the envelope of stimulation signal.

In the case of tTIS, PLV is computed between the LFP and the envelope of the stimulation signal. The heat map representing phase entrainment (Figure 4B) notably exhibits differences between tACS and tTIS, particularly the absence of an Arnold tongue in the harmonic frequency of stimulation in tTIS.

Significantly, the amplitude required to elicit comparable effects to tACS is notably higher in tTIS. Considerably higher amplitude—approximately 2000 pA—is needed to effectively stimulate the network when *f*_*m*_ aligns with the intrinsic frequency. This requirement represents a hundredfold increase compared to the minimum amplitude necessary for achieving tACS phase entrainment at a 9 Hz stimulation. This substantial amplitude difference underscores the considerably higher requirement in tTIS to achieve effects similar to tACS.

Computations were conducted to monitor frequency entrainment akin to tACS, determining the dominant frequency within the LFP (Figure 4C) and calculating the power associated with this specific frequency (Figure 4D). The shift in the dominant LFP frequency signifies the transition from intrinsic oscillation frequency to the stimulation frequency, indicating frequency entrainment. Notably, the absence of Arnold tongue at stimulation frequencies equivalent to the harmonics of the intrinsic frequency can be observed more prominently in tTIS compared to tACS. Unlike tACS, when the stimulation frequency matches the harmonics of the intrinsic frequency, the dominant frequency of the LFP aligns with the stimulation frequency in tTIS. For instance, at a stimulation frequency of 18 Hz, we initially witness no frequency modulation for lower amplitudes. However, as the stimulation amplitude increases, frequency modulation emerges, eventually resulting in the dominant frequency of LFP aligning with 18 Hz.

Similar to tACS, the regions exhibiting frequency modulation are classified into three zones in tTIS (Figure 4C) including: frequency entrainment (indicated by A), non-entrainment (indicated by C) and frequency entrainment aligned with the subharmonic of the stimulation frequency (indicated by B).

The delineating yellow demarcation between area A and B is determined by computing the power within the subharmonic of the stimulation frequency (fm/2) as depicted in Figure 4F.

Distinct entrainment patterns would appear at different beat frequencies. At beat frequency less than 15 Hz, increasing the stimulation amplitude leads to an increase in PLV and power at dominants frequency of LFP. Moreover, the dominant frequency shifts from the intrinsic frequency to match the stimulation frequency.

However, frequencies higher than 15 Hz manifest a distinct pattern. For instance, as the stimulation amplitude increases at 30 Hz, the dominant frequency of the LFP initially aligns with the stimulation frequency. During this phase, the PLV increases to 0.2, accompanied by a slight rise in the power of the dominant frequency. Subsequently, with further increases in stimulation amplitude, the network loses its entrainment. By placing the dominant frequency in resonance with half of the stimulation frequency, entrainment is reestablished and the dominant frequency once again aligns with the stimulation frequency. In this scenario, the PLV increases to 0.5, while the power in the dominant frequency band demonstrates a clear rise.

Upon computing the power within the stimulation (Figure 4G) and intrinsic oscillation bands (Figure 4E), it becomes evident that resonance solely arises when applying a stimulation frequency equivalent to the intrinsic oscillation. The presence of the Arnold tongue within the stimulation power band signifies an increase in power within this band with escalating stimulation amplitude. Notably, the findings indicate that the farther the stimulation frequency is from the network’s intrinsic frequency, the greater amplitude is required to achieve the same results.

### 3.3 Multi scale model

The generated field distribution using 1 mA current for each electrode, is depicted in Figures 1B–D. In Figure 1C, the maximum envelope amplitude $\left|{\vec{E}}_{A\,M}^{m\,a\,x}\,\left(\vec{r}\right)\right|$ reaches its highest intensity at the interference center. Conversely, (Figure 1D) reveals the normalized value of the maximum envelope amplitude (*N*_*MD*) beyond the interference center. This indicates that while the maximum envelope amplitude peaks at the interference center, the ratio of the envelope and stimulation signal amplitudes remain steady across different areas. Furthermore, moving closer to the electrodes increases the stimulation signal’s amplitude but reduces its envelope amplitude. Consequently, understanding how the distance from the electrodes to the interference center affects the spatial resolution of the tTIS method is crucial. Section 2.6 elaborates on the methodology employed to assess the effects of tTIS neuromodulation within the spherical head model. To comprehensively observe these effects, a stimulation frequency of 13 Hz was chosen, utilizing two pairs of electrodes operating at 1 kHz and 1013 Hz, respectively. This chosen stimulation frequency enables the comprehensive examination of frequency entrainment alongside the analysis of amplitude and phase entrainment. Determining the necessary current intensity for the electrodes involved referring to the PLV heat map in Figure 4B, which identified 4400 pA as *I*_*stim–th*_. This value signifies the minimum amplitude required for the modulated stimulus signal to impact the network at the interference center. As a result, a current of 2200 pA is applied to each pair of electrodes, yielding a total current of 63.5 mA as *I*_*electrode*−*th*_ in each electrode pair and generating an equivalent electric field of 23 V/m.

**FIGURE 5:**
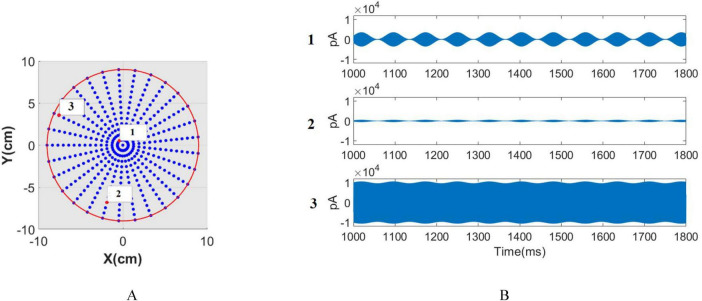
Network distrubation. **(A)** The network distribution, where each point represents a network consisting of 80 pyramidal neurons and 20 inhibitory neurons. **(B)** stimulation signals corresponding to the network highlighted with a red circle in panel **(A)**.

Through applying the*I*_*electrode*−*th*_ to individual pairs of electrodes, critical parameters like ${\vec{E}}_{1}$, ${\vec{E}}_{2}$, and$\left|{\vec{E}}_{A\,M}^{m\,a\,x}\right|$ can be computed. The time signals allocated to each (x, y, z) coordinate, as specified in section 2.6, serve as stimulus signals for individual networks. Consequently, each network receives a unique input signal varying in both amplitude and modulation depth. Each network model resides at a single point in the 3D space, and the input current values are calculated via the e-field at that specific (x, y, z) point. The network models are distributed on a regular grid within the spherical space. The network distribution is depicted in Figure 5A, where each point represents a network consisting of 80 pyramidal neurons and 20 inhibitory neurons. The stimulation signals corresponding to the network highlighted with a red circle in Figure 5A are shown in Figure 5B. These stimulation signals differ in both amplitude and modulation depth depending on the network’s spatial location. Notably, Point 2 has a high modulation depth but low amplitude, while Point 1 has a high modulation depth but greater amplitude compared to Point 2. Point 3, on the other hand, shows low modulation depth but high amplitude. The subsequent calculation of phase, amplitude, and frequency entrainment aims to evaluate the efficacy of tTIS within the interference center and across the span between electrodes and the interference center.

**FIGURE 6:**
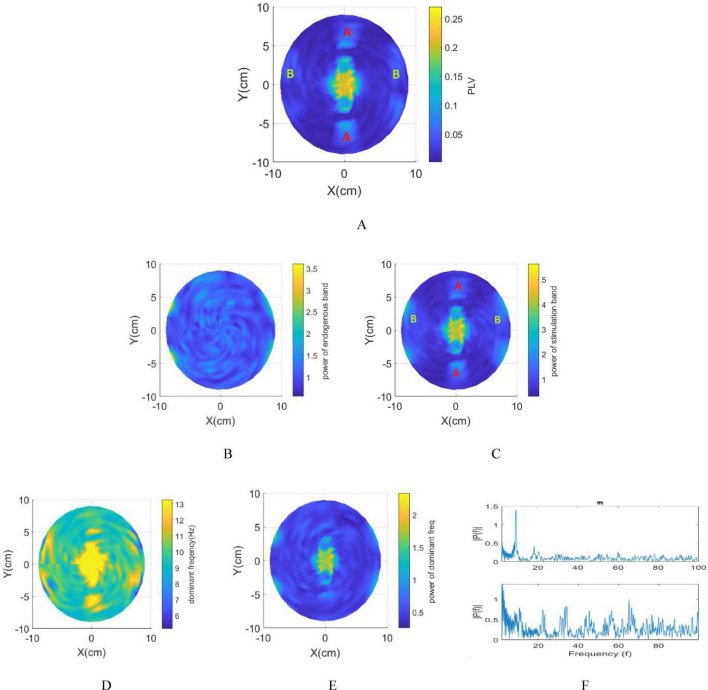
Integration of spherical head model and network model. **(A)** Phase entrainment heat map using 13 Hz as the stimulation frequency. **(B)** Amplitude entrainment: Power within the intrinsic frequency band. **(C)** Amplitude entrainment: Power within the stimulation frequency band. **(D)** Frequency entrainment heat map. **(E)** Power of dominant frequency in LFPs. **(F)** Top: FFT representation of the LFP depicting intrinsic oscillations (without stimulation). Bottom: FFT representation of LFP recorded in the proximity of the electrodes (B region).

The resulting brain phase entrainment map, depicted in Figure 6A, indicates an increased PLV not only at the interference center but also in regions denoted as A and B. Region A demonstrates sensitivity to stimulation, evidenced by a larger *N*_*MD* within this area.

However, due to the lower envelope amplitude of the stimulation signal in region A compared to the interference center, the PLV in this region is also smaller than in the interference center. Conversely, Area B, located in the superficial area of the brain and closer to the electrodes, exhibits a notable rise in PLV despite the lower amplitude of the envelope signal. This increase is attributed to the substantial stimulation amplitude applied in this area. Using Digimizer, the ratio of the stimulated brain area to the total brain volume in the depth of the brain is calculated. This percentage is 4.41% based on macroscopic modeling, while based on multidimensional modeling focusing on phase entrainment, this ratio decreases to 2.4%.

The power within the stimulation band (Figure 6C) notably increases near the electrodes (region A) and the interference center (region B). This heightened power, seen across both the 9 Hz and 13 Hz bands in response to 13 Hz stimulation frequency, arises from the high-amplitude, high-frequency stimulation near the electrodes. Despite this increased power within region A, there’s a noticeable lack of frequency entrainment except for a small area (Figure 6D). While Area A shows amplitude modulation, characterized by an increase in power within the stimulation band, the dominant frequency doesn’t shift. In this area, the dominant frequency of the LFP consistently remains at 9 Hz. Notably, the limited area in region A demonstrating frequency entrainment could have unintended consequences depending on the application of tTIS, potentially leading to adverse effects.

frequency modulation (Figure 6D) illustrates effectiveness of stimulation using tTIS in the interference center. The blue lines below the electrodes signify a transition in the dominant frequency to 5 Hz, albeit with minimal power observed at this frequency (Figure 6E). This state emerges when the modulation depth approaches zero, causing the network to lose its synchrony due to the high amplitude stimulation in this specific region. The LFP under this condition is illustrated in Figure 6F. The observed increase in power across all frequencies during stimulation confirms our previous results.

Therefore, as depicted in Figure 6, at the interference center, the dominant frequency equals the stimulation frequency and its power is maximized. Moving away from the interference center results in a gradual diminishment of the power of the dominant frequency until the stimulus is no longer effective in entraining the network’s frequency. Subsequently, the dominant frequency reverts to the intrinsic frequency of the network, i.e., 9 Hz. In these areas, despite the absence of frequency modulation, an increase in power within the stimulation band is observed, while the power in the endogenous band remains constant, indicating the impact of stimulation in some of these non-target areas.

In the depth of the brain, the ratio of the stimulated brain area to the total brain volume demonstrates a noteworthy increase in the power of the stimulation band, comprising 2.41% of the brain model. Additionally, 9.6% of this area experiences frequency entrainment, signifying a 26% higher power than the remaining entrained frequencies.

### 3.4 Result discussion

By employing the E-I network and varying the frequency and amplitude of tTIS, this study investigates the network’s response, focusing on three crucial parameters: phase, amplitude, and frequency entrainment. These parameters are derived from LFP estimation obtained from the network. Expanding on previous research suggesting that neuronal response corresponds to the envelope frequency of stimulation signals (f_*m*_), this study also investigates these parameters for sinusoidal stimulation (tACS) resembling the stimulation frequency utilized in tTIS. Figures 3, 4 reveal distinct network responses between pure sinusoidal stimulation and tTIS methodologies, attributed to the high-frequency component present in the tTIS signal. Consequently, based on Izikvich neuron network modeling, definitive conclusions about responsiveness of neurons to high frequencies cannot be drawn. High-frequency stimulation elicits various neuronal reactions, including neuron conduction blocking, synapse fatigue, facilitation, and temporal summation, as detailed in prior literature (Neudorfer et al., 2021). The observed low-pass filtering effect in the neuron membrane (Reato et al., 2010), coupled with kHz frequency components in the stimulus signal, underscores the need for higher amplitude in tTIS compared to tACS to achieve comparable outcomes. This aligns with earlier research investigating tTIS through axon or neuron modeling (Xiao et al., 2019; Mirzakhalili et al., 2020; Esmaeilpour et al., 2021; Howell and McIntyre, 2021).

Considering the neuronal membranes role in low-pass filtering, the necessity for higher intensity in neuronal response and microscopic modeling becomes evident, as macroscopic modeling does not account for attenuation in stimulation amplitude (Rampersad et al., 2019; Huang and Parra, 2019). Additionally, macroscopic models outline where and how temporal interference occurs. They also highlight the stimulation signals envelope amplitude as the driving force, forming the basis for ROI calculations (Karimi et al., 2019; Rampersad et al., 2019). Although these calculations are vital for ROI determination, the variation in stimulation effects observed between macroscopic and microscopic approaches highlights the imperative nature of investigating the stimulation effect initially from a neuronal perspective and subsequently extrapolating it to the entire brain using macroscopic models. Therefore, this paper initially explores the neuronal effects before extrapolating and calculating these effects in the whole brain.

### 3.5 Comparison between tACS and tTIS

A comparison between (Figure 3A) and (Figure 4B), illustrating phase entrainment for tACS and tTIS respectively, reveals a notable disparity in the required current intensity with tTIS necessitating a significantly higher intensity than tACS. In the case of tACS, the phase entrainment map exhibits feature associated with the forced oscillator, including entrainment to external frequencies, harmonic entrainment where the natural frequency synchronizes not only with the exact frequency of the external input but also with frequencies that are integer multiples (harmonics) of their natural frequency. This response occurs within a specific range, and nonlinear effects can lead to behaviors such as subharmonic or superharmonic entrainment, where the oscillator synchronizes at frequencies that are fractions or multiples but not exact harmonics of its natural frequency. Meanwhile, superharmonic entrainment is observed in both stimulations, while harmonic entrainment is not observed in tTIS stimulation. Furthermore, superharmonic entrainment is different in tACS and tTIS (Figure 3G) and (Figure 4F). The superharmonic entrainment for tACS corresponds to a forced oscillator that entrains the 9 Hz band for a stimulation frequency of 18 Hz, while for tTIS there is superharmonic entrainment for a stimulation frequency more than 15 Hz.

In summary, the differences in entrainment behavior between tACS and tTIS likely arise from the oscillator’s response range, nonlinear effects introduced by the kHz stimulation, frequency discrimination capabilities, resonance properties, and the complexity of the systems dynamics. These factors collectively contribute to the observed differences in entrainment at harmonics and subharmonics of the natural frequency.

Also, the frequency entrainment displays distinctive pattern maps for tACS and tTIS (Figure 3C) and (Figure 4C). Specifically, regarding tACS, three zones are identifiable: non-frequency entrainment, frequency entrainment in alignment with the stimulation frequency, and frequency entrainment when the stimulation frequency corresponds to the harmonic of the intrinsic frequency. Conversely, the observed zones for tTIS include: frequency entrainment in alignment with the stimulation frequency, non-entrainment, and frequency entrainment aligned with the subharmonic of the stimulation frequency. Moreover, the spatial distribution of these areas varies significantly for each stimulation method.

### 3.6 Spatial resolution and modulation depth

The spatial resolution of tTIS is determined via computational analysis using neuronal networks and brain field distribution. This analysis assesses the impact of stimulation frequency and amplitude, with a specific focus on modulation depth to calculate spatial resolution and understand potential side effects. Prior methods for determining spatial resolution relied on macroscopic modeling, employing criteria like FWHM (Grossman et al., 2017) or threshold methodologies (Rampersad et al., 2019), without accounting for neuronal circuit impacts.

Leveraging neural network behavior and electric field distribution allows the generation of a brain map, facilitating the assessment of stimulation effects at a consistent 13 Hz stimulation frequency. This evaluation of spatial resolution stimulation involves assessing phase, amplitude, and frequency entrainment criteria.

The findings underscore modulation occurring not only in deep brain areas but also on the surface. Specifically, there’s a noticeable rise in power within both the intrinsic oscillation band and the stimulation band beneath the electrodes, attributed to the high stimulus signal amplitude. Moreover, these areas lack a dominant frequency for LFP, leading to increased power across all frequency bands due to very low modulation depth and high stimulation amplitude in this particular region.

In non-target zones, despite high *N*_*MD* values, augmented PLV, and increased power within the stimulation band, the effects of tTIS remain more pronounced in deep brain areas. This partial frequency entrainment and other observed forms of entrainment suggest that while tTIS can influence deep brain structures, its overall effectiveness is limited by non-selective stimulation and prominent surface effects, which could reduce its precision in deep brain targeting.

### 3.7 Comparison to other recent studies

In this work, the neuromodulation effects of tTIS stimulation in parametric space are investigated. The Arnold tongue in the entrainment map for tACS is consistent with previous studies, which indicate that synchronization can also occur at harmonics and subharmonics of the endogenous frequency (Vogeti et al., 2022), in addition to the endogenous frequency itself. While prior research has investigated the effect of tTIS on gamma oscillation at a beat frequency of 5 Hz, our study provides insights into the entrainment map with different features, showing that LFP is affected by tTIS. Although their results suggest that a 60 V/m electric field (167 mA) is required to achieve significant neuromodulatory effects (for a beat frequency of 5 Hz and a carrier frequency of 1 kHz), our findings, detailing the effects of tTIS on phase, amplitude, and frequency entrainment, suggest that the minimum amplitude needed for a neuromodulatory effect corresponds to a 20 V/m electric field at the interference center (for a beat frequency of 9 Hz and a carrier frequency of 1 kHz). Additionally, our results are consistent with other studies that observed only a minimal increase in neuron firing rate with tTIS (at a beat frequency of 10 Hz and a carrier frequency of 1 kHz) with an electric field of 10 V/m (Howell and McIntyre, 2021). These findings, based on neuron modeling in tTIS, suggest that tTIS may not be practical due to the substantial difference between the electric fields induced by an amplitude of 2 mA (equivalent to 0.1–0.2 V/m) and the threshold for effective stimulation. This is corroborated by recordings from single neurons in non-human primates, which show that tTIS reliably alters only the timing of spiking activity without affecting its rate and remains approximately 80% less effective than other non-invasive brain stimulation techniques, making it unlikely to induce widespread neuronal entrainment (Vieira et al., 2024) at an amplitude of 2 mA.

Moreover, multi-scale modeling suggests that, in addition to stimulating deep brain regions, superficial areas are also affected by tTIS. According to axon modeling (Mirzakhalili et al., 2020), axons in superficial areas are blocked and exhibit tonic firings. E-I network models indicate that superficial areas experience static modulation, as evidenced by measuring the mean power in the gamma band (Esmaeilpour et al., 2021). However, our study shows phase and frequency entrainment in non-target areas, indicating potential side effects of tTIS alongside its capability for deep brain stimulation.

### 3.8 Limitations of the model

While our single-layer spherical head model provided an adequate initial analysis, we recognize the necessity of utilizing more accurate head models to better understand the effects of tTIS and tACS. Further experimental research and refined simulations are essential for optimizing these neuromodulation techniques for therapeutic use. Our current findings offer a preliminary exploration into the feasibility of tTIS and tACS for network modulation. Advancing our modeling techniques will enhance the reliability and relevance of our results, ultimately contributing to a deeper understanding of these neuromodulation methods and their potential applications in human studies.

Our current modeling does not account for factors such as synaptic plasticity (Qi et al., 2024) and other neuromodulatory effects like burst rate, burst duration, and interspike interval (ISI), which have been shown to be influenced by tTIS in recent studies using in vitro ‘TIS on a chip’ platforms (Ahtiainen et al., 2024). Incorporating these factors into computational network models could provide a more comprehensive understanding of tTIS’s impact on neural activity.

Furthermore, the application of our computational model to real-world scenarios is limited by the simplifications and assumptions inherent in the model. Realistic head models that incorporate individual anatomical and physiological variations are crucial for more accurate predictions of tTIS and tACS effects. Future studies should integrate advanced brain models, such as the multi-layer sphere model (Karimi et al., 2019) or those derived from MRI images (Huang and Parra, 2019; Violante et al., 2023), to enhance the applicability of the findings. These models should also consider the heterogeneity of brain tissue properties and the influence of other neuromodulatory effects. Overall, while our study provides valuable insights into the mechanisms of tTIS and tACS, further experimental validation and refinement of the computational models are necessary to fully understand their potential and limitations in clinical settings.

Moreover, while our current modeling employs the Izhikevich neuron model, it may not fully capture suprathreshold mechanisms of action, such as tonic firing or conduction block, which are often missed in network models but are critical factors in determining tTIS selectivity, especially in brain regions near the electrodes. Research has identified conduction block as a potential side effect of tTIS (Mirzakhalili et al., 2020), highlighting the need for models that can more accurately reflect these effects. To better understand these mechanisms and improve model accuracy, incorporating alternative neuron models, such as the Hodgkin-Huxley model (Cao et al., 2020), could be beneficial. The Hodgkin-Huxley model, with its detailed representation of nonlinear membrane properties, would provide a more comprehensive examination of the diverse effects of stimulation at the single-neuron level, particularly in regions susceptible to suprathreshold effects. Additionally, considering factors like neuron dimensions and orientation is crucial for effective stimulation. Finally, integrating insights from human studies (Ma et al., 2021; von Conta et al., 2022) will be essential in fully understanding and optimizing tTIS for effective and selective deep brain stimulation.

## 4 Conclusion

The limited depth of human research has posed challenges in effectively stimulating deep brain targets via tTIS, aiming to stimulate these regions without affecting surface areas. This study explores the parameters influencing tTIS effectiveness, particularly examining the differences in modulation depth between deep and surface areas. Deep regions experience stimuli with greater modulation depth but lower amplitude compared to surface regions. This variation in modulation influences stimulation efficiency, depending on the complex interaction between the brain’s intrinsic oscillations and the stimulation signal.

Given the constraints on applied current intensity in human transcranial brain stimulation, a primary objective of electrical stimulation—especially alternating current—is to modulate brain oscillations. Moreover, heightened sensitivity to alternating stimulation is noted in active neurons and concurrent network activities (Radman et al., 2007; Liu et al., 2018; Vosskuhl et al., 2018; Howell and McIntyre, 2021). This interplay between intrinsic brain oscillations and the stimulus signal is specifically examined within alpha oscillations. By employing the E-I network and varying the frequency and amplitude of tTIS, this study investigates the network’s response, focusing on three crucial parameters: phase, amplitude, and frequency entrainment. These parameters are derived from LFP estimations obtained from the network.

This study also investigates the distinct network responses between sinusoidal stimulation (tACS) and temporal interference stimulation (tTIS), highlighting key differences attributed to the high-frequency component in tTIS. The analysis reveals that tTIS requires a significantly higher current intensity compared to tACS to achieve comparable outcomes due to the low-pass filtering effect of the neuron membrane. The study emphasizes the necessity of examining stimulation effects at the neuronal level before extrapolating to the whole brain, noting variations in phase, amplitude, and frequency entrainment between tACS and tTIS. The findings highlight that while tTIS effectively modulates deep brain areas, it also affects surface regions, presenting challenges in achieving precise selectivity. Additionally, a high stimulus signal amplitude is required to attain the desired effects in the targeted zones.

## Data Availability

The original contributions presented in the study are included in the article/supplementary material, further inquiries can be directed to the corresponding author.
